# Unstented large fenestration for close target vessel ostia: Long-term follow-up

**DOI:** 10.1177/17085381251328062

**Published:** 2025-03-19

**Authors:** Lorenzo Torri, Giuseppe Panuccio, Petroula Nana, Jose Ignatio Torrealba, Tilo Kölbel

**Affiliations:** German Aortic Center, Department of Vascular Medicine, 37734 University Medical Center Eppendorf, Hamburg, Germany

**Keywords:** Aorta, endovascular repair, celiac trunk, long-term follow-up, penetrating aortic ulcer

## Abstract

**Purpose:**

To report the 10-year follow-up of a patient managed with a custom-made fenestrated endograft, including a large fenestration for the preservation of a celiac trunk (CT) anatomic variation.

**Case report:**

In 2014, a 75-year-old female was treated endovascularly for descending thoracic and visceral aortic pseudoaneurysms (PA). Due to a celiac trunk (CT) anatomic variation, consisting of separate origins of the hepatic and splenic artery, a custom-made four-fenestrated endograft was planned, including a large fenestration (18 × 10 mm) for CT preservation. Balloon-expandable covered stents bridged all target vessels (TVs), except the large fenestration, which was left unstented. Imaging at 10 years showed patency of TVs, no signs of instability or device migration and complete PA exclusion.

**Conclusion:**

Using a patient-specific device for complex endovascular aortic repair provided favorable extended follow-up outcomes. An unstented large fenestration remained patent during 10 years of follow-up. This case highlights the importance of individualized approach in complex aortic pathologies.

## Introduction

Penetrating aortic ulcers (PAUs) are classified among acute aortic syndromes (AAS), despite that they often represent an incidental imaging finding.^1^ Their evolution is not easily predictable, as they may remain stable, enlarge, or progress to intramural hematoma (IMH), dissection, pseudoaneurysm (PA), or even rupture.^1^

Fenestrated and branched endovascular aortic aneurysm repair (f/bEVAR) has become the standard of care for many thoracoabdominal aortic pathologies. However, several challenges with visceral vessels remain unresolved. These challenges are usually related to early division of target vessels, anatomic variations and target vessels with closely located origins. This fact lead in many cases to more extensive coverage and the need of customized endografts, as the availability of off-the-shelf thoracic and thoracoabdominal devices, accommodating to target vessel variations, remains limited. Solutions, including double barrel stenting into the same fenestration or branch, bidirectional branches, large unstented fenestrations or even target vessel sacrifice, have been proposed; with the experience remaining limited, and their durability being questionned.^2,3^

This case report aimed to present the 10-year follow-up of a patient with pseudoaneurysms of the visceral and descending thoracic aorta managed with a custom-made endograft, including a large fenestration for the preservation of a celiac trunk (CT) anatomic variation.

A written consent has been assigned by the patient, confirming agreement to the publication of the case details and images.

## Case report

### Case description

**Figure 1.**
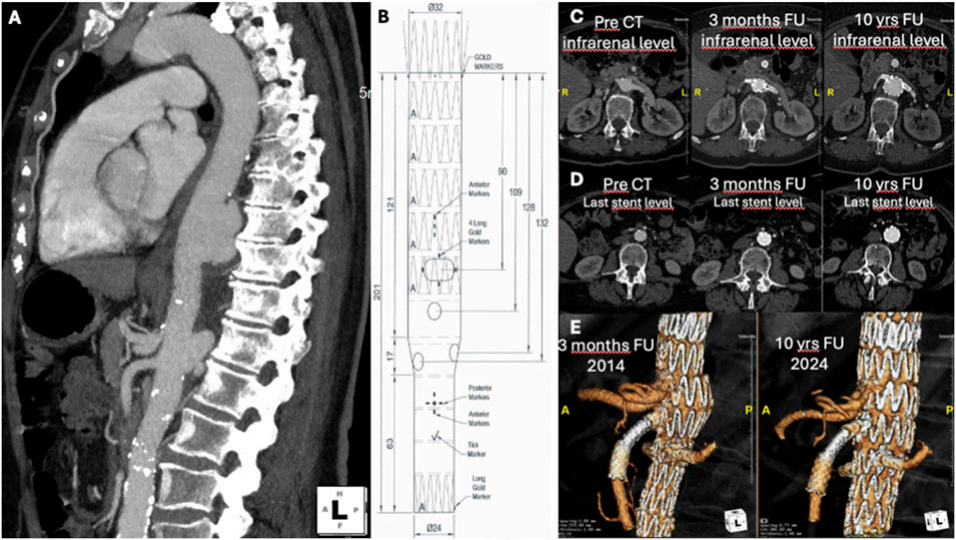
Case images. (A) Multi Intensity Projection (MIP) Computed Tomography Angiography (CTA) in sagittal view that shows the thoracic and visceral PAUs pseudoaneurysm; (B) fEVAR graft plan; (C-D) axial view of CTA at different time of the aortic level with the bigger diameter changes; (C) infrarenal level aortic diameter changes; (D) last stent level aortic diameter changes; (E) 3D reconstruction of the graft at 3 months follow-up (FU) and 10 years FU.

In 2014, a 75-year-old female, with a history of hypertension, was admitted to the hospital due to two symptomatic pseudoaneurysms; one located in the distal descending thoracic aorta (40 × 20 mm) and another at the posterior wall of the visceral aorta (15 × 30 mm) (Figure 1A). The mean aortic diameter proximal and distal to the pseudoaneurysms was 25 mm and 20 mm, respectively. In addition, an anatomic variation of the CT with a very close (1 mm) but separate origin of the hepatic and splenic artery was present.

As a synchronous endovascular repair of both pseudoaneurysms would demand a long aortic coverage and increase the risk of spinal cord ischemia a two stage approach was decided. The first step would include the coverage of the bigger thoracic pseudoaneurysm using a standard thoracic device, with landing proximal to the CT while for the second step, an urgent custom-made fenestrated endograft would be designed and shipped within 4 weeks.

### Graft plan

The custom-made device (CMD) was designed to exclude the visceral pseudoaneurysm and land proximally into the previous thoracic device. A tapered configuration was chosen to adapt to the aortic diameter difference between the thoracic and abdominal aorta, with a total length of 201 mm (Figure 1B) and a total aortic coverage of 277 mm. The endograft included four fenestrations; three strut-free fenestrations for the superior mesenteric (SMA; 8 × 8 mm) and the renal arteries (6 × 8 mm) and one large (18 × 10 mm) fenestration, for the preservation of both the hepatic and splenic artery. This large fenestration had stent-struts crossing and was not planned to be stented.

### Procedures

Both operations were performed in a hybrid operating room with a fixed imaging system and the patient in supine position under general anesthesia. Accesses were achieved with bilateral common femoral cut downs. Systemic heparinization at 100 IU/kg with a target activated clotting time >250 s was obtained.

In the first procedure, a 36 × 157 mm ZTEG (Cook Medical, Bloomington, IN, USA) was advanced from the right side and deployed in standard fashion i to cover the thoracic pseudoaneurysm.

During the second procedure, after 5 weeks, the CMD, loaded in a 22F sheath, was advanced from the right common femoral artery. Using the left femoral access, all fenestrations and target vessels (TVs) were catheterized, except the large fenestration. The reducing ties were released and bridging of the three target vessels (TVs) was performed, using balloon-expandable covered stents [renal arteries: both with 7 × 22 mm Advanta V12, (Atrium Medical Corporation, NH, USA) and SMA: 8 × 38 mm Advanta V12), followed by flaring with a 10 mm balloon. The large fenestration for the common orifice of the hepatic and splenic artery was left unstented. The completion angiography confirmed pseudoaneurysms exclusion and perfusion of all TVs.

The patient had an uneventful postoperative course. The pre-discharge CTA showed no endoleak and confirmed the findings of the intra-operative angiography. Follow-up at 3 months confirmed TV patency, including the celiac variation, and exclusion of both pseudoaneurysms (Figure 1C–E).

### Long term follow-up

The patient was followed at 1, 3, 6 and 10 years using computed tomography angiography. Imaging evaluation showed pseudoaneurysms’ regression, with intact sealing zones (Table 1). No graft migration was observed after evaluating three different measurements: the distance from the TVs ostium to the proximal graft stent marker, the distance from the TVs’ ostium to the distal graft stent marker, and the distance between TVs (Table 2). No bone structures were used as markers for follow-up measurements, as changes in vertebral anatomy due to osteoporosis were noted. All TVs remained patent without stenosis or endoleak. No significant difference was detected between the 3-month and 10-year CTA. Similarly, no bridging stent fracture or migration was noted, after estimating the distance between the fenestration and the distal point of the bridging stent.

**Table 1.** Measurement (in mm) of diameters at different levels of aorta and visceral vessels and difference in diameters (in mm) over time.

Diameter level	Pre-CT (mm)	3 months FU (mm)	10 yrs FU (mm)	Pre-CT versus 3 months FU (diameter difference mm)	Pre-CT versus 10 yrs FU (diameter difference mm)	3 months FU versus 10 yrs FU (diameter difference mm)
Proximal stent	32	35	40	3	8	5
Thoracic PAU	47	35	35	−12	−12	0
Celiac trunk PAU	33	30	35	−3	2	5
Superior mesenteric artery	29	30	35	1	6	5
Infrarenal	22	22	29	0	7	7
Distal stent	21	22	29	1	8	7
Aortic bifurcation	19	19	20	0	1	1
Right renal artery (5 mm post stent)	8	8	7	0	−1	−1
Right renal artery (intrastent)	—	8	8	—	—	0
Left renal artery (5 mm post stent)	7	5	5	−2	−2	0
Left renal artery (intrastent)	—	8	8	—	—	0
Superior mesenteric artery (5 mm post stent)	6	7	8	1	2	1
Superior mesenteric artery (intrastent)	—	10	11	—	—	1
Hepatic artery origin	8	8	7	0	−1	−1
Hepatic artery distal (3 cm)	8	8	6	0	−2	−2
Splenic artery origin	8	8	8	0	0	0
Splenic artery distal (3 cm)	7	7	5	0	−2	−2

**Table 2.** Graft migration measurement (in mm), to compare measurement at 3 months FU and 10 years FU.

Graft migration measurements	3 months FU (mm)	10 yrs FU (mm)
Celiac trunk to proximal stent graft distance	182	181
Celiac trunk to distal stent graft distance	107	107
Celiac trunk to RRA distal stent distance	58	57
Superior mesenteric artery to proximal stent graft distance	202	201
Superior mesenteric artery to distal stent graft distance	88	88
Superior mesenteric artery to celiac trunk origin distance	16	16
Superior mesenteric artery stent length inside vessel	46	46
Right renal artery to proximal stent graft distance	222	221
Right renal artery to distal stent graft distance	69	69
Right renal artery stent length inside vessel	12	12
Left renal artery to proximal stent graft distance	225	224
Left renal artery to distal stent graft distance	65	65
Left renal artery stent length inside vessel	16	16

## Discussion

The use of a single large fenestration for the hepatic and splenic arteries in this specific case was driven by anatomical and technical considerations, and was related to 10-year durability, showing that customized solutions may provide optimal outcomes in patients undergoing complex endovascular repair.

The major concern in this case was the preservation of the almost common orifice of the hepatic and splenic artery, which was achieved using a large unstented fenestration. Alternatively, a close distance between TV ostia in f/bEVAR could be resolved with the use of two separate fenestrations. For the Cook Zenith platform, as well as the Anaconda custom-made device (Terumo Aortic), which was also available in 2014, the minimum distance between two fenestrations was 2 mm, according to manufacturer’s instructions. This approach was rejected in this case due to the high risk for misalignment, difficult catheterization and further, compression of the two parallel bridging stents.

Another option for both TV preservations would be the use of a smaller strut-free fenestration, in addition to the double barrel stenting technique. However, this approach would carry a risk of bridging stent compression and potential occlusion, along with a higher (4–7%) risk for type IIIc endoleak, even within the early follow-up.^2,4,5^ If the patient was managed today, after the advancements in technique and technology of the last years, an alternative would be the use of bidirectional inner branches, which have shown encouraging early results^6^ and would have allowed selective stenting of the splenic and hepatic arteries despite their very close origins; however, this technology was not available a decade ago.

Again, patient’s specific anatomy and distances of TVs would play a major role in endograft-design making and probably, a single large fenestration with unstented TVs would be the only viable solution for this patient. However, the presence of a single large fenestration with crossing stent struts could complicate future endovascular solutions in case of disease progression at the celiac trunk level, making stenting at this level necessary and exposing the patient to the potential complications already described, which could become even more likely due to the presence of stent struts.

Stenting vs non-stenting of the CT in complex endovascular procedures is still a topic of controversy, especially in terms of long-term durability.^5,7,8^ Unstented CT fenestrations have been associated with clinically silent TV instability, mainly driven by CT occlusion rather than reinterventions or intestinal ischemia.^8^ Even if the CT is occluded during follow-up, it is unlikely to lead to clinically significant consequences.^7^ Ten-year follow-up provides important data about the stability of both TVs and endograft in this case, despite that the process of aging led to alterations of both bone and vascular structures.

The published literature provides limited information about the outcomes of CMDs in patients with PAUs or pseudoaneurysms; however, current recommendations suggest endovascular management.^1,9^ The main reason for this gap in evidence is that patients with PAUs and pseudoaneurysms are usually included in larger dissection or degenerative aneurysm cohorts. In addition, long-term data on f/bEVAR are lacking and most studies provide information only up to 5 years; studies that extend to 10 years or more are limited, making it difficult to assess the full durability and efficacy of f/bEVAR over the long-term. Under this spectrum, this case report might provide a useful insight in the very long-term follow-up of f/bEVAR. However, as previously shown in the literature, the impact of patient’s surveillance compliance on f/bEVAR outcomes remains unclear.^10^ Thus, potential reporting bias should be taken into consideration when assessing the findings of this report. Cohort studies are needed to reassure the long-term outcomes of f/bEVAR and better clarify the safest and more durable option for target vessel preservation.

## Conclusion

A patient-specific custom-made endograft, including a large unstented fenestration for the hepatic and splenic artery, provided favorable extended follow-up and confirmed the durability of the approach in this case. This case highlights the importance of individualized approach in complex aortic pathologies and the need for long-term data in patients managed with complex endovascular procedures.
